# Western Cold and Flu (WeCoF) aerosol study – preliminary results

**DOI:** 10.1186/1756-0500-7-563

**Published:** 2014-08-23

**Authors:** Eric Savory, William E Lin, Karin Blackman, Matthew C Roberto, Lauren R Cuthbertson, James A Scott, Samira Mubareka

**Affiliations:** Department of Mechanical & Materials Engineering, The University of Western Ontario, 1151 Richmond Street North, London, N6A 5B9 Canada; Division of Occupational and Environmental Health, Dalla Lana School of Public Health, University of Toronto, 223 College Street, Toronto, M5T 1R4 Canada; Department of Biological Sciences, Sunnybrook Research Institute, 2075 Bayview Avenue, Toronto, M4N 3M5 Canada

**Keywords:** Cough, Cold, Influenza, Particle Image Velocimetry, Airflow sampling, Bioaerosol

## Abstract

**Background:**

Influenza virus is responsible for annual deaths due to seasonal epidemics and is the cause of major pandemics which have claimed millions of human lives over the last century. Knowledge about respiratory virus transmission is advancing. Spread is likely through the air, but much work remains to be done to characterize the aerosols produced by infected individuals, including viral particle survival and infectivity. Although coughs have been characterized, little work has been done to examine coughs from infected individuals. The WeCoF project aims at providing evidence to support prevention measures to mitigate person-to-person influenza transmission in critical locations, such as hospitals, and during pandemics.

**Findings:**

A novel experimental cough chamber facility – the FLUGIE – has been developed to study the far-field aerodynamics and aerosol transport of droplets produced by the coughs from humans naturally-infected with influenza. The flow field of each cough is measured using Particle Image Velocimetry (PIV). A preliminary study involving 12 healthy individuals has been carried out in order to quantify the strengths of their coughs at a distance of 1 m from the mouth. The spatially averaged maximum velocity was determined and the average value was 0.41 m/s across 27 coughs of good data quality. The peak value of velocity was also extracted and compared with the average velocity.

**Conclusions:**

Preliminary results show that there is significant air motion associated with a cough (on the order of 0.5 m/s) as far away as 1 m from the mouth of the healthy person who coughs. The results from this pilot study provide the framework for a more extensive participant recruitment campaign that will encompass a statistically-significant cohort.

## Findings

### Introduction

We have gained extraordinarily detailed knowledge in the past decade about the molecular nature of influenza virus and other respiratory viruses yet surprisingly little is known about how respiratory viruses are transmitted from person to person. Mathematical modelling of households, containing infected individuals, showed aerosol transmission to be more significant than contact transmission for influenza virus and that airborne transmission may be a significant contributor [1, 2]. Recent reviews of the literature support this important possibility [3, 4].

Aerosols consist of particles in a range of sizes. Traditionally droplets of >5 μm diameter have been implicated in short range (<1 m) spread, and droplet nuclei of <5 μm are believed to be responsible for longer range or airborne transmission (>1 m) [5]. However, larger-sized particles may be responsible for wider pathogen spread depending on other factors. For example, particles of the same size may travel different distances, depending on the velocity of the jet propelling them [6]. These particle dynamics remain undefined in the clinical setting, and the implications are significant from an infection prevention and control perspective. In addition to its effect on dispersion, particle size has implications for the inhaling host. Larger particles (>10 μm) will be deposited by impaction in the upper respiratory tract and smaller particles (0.003-5 μm) may penetrate the tracheobronchial and alveolar regions, principally through sedimentation and diffusion [7]. By studying the aerosols produced by infected individuals, we hope to precisely characterize how long virus infectivity persists in suspended aerosol droplets of various sizes. Recent work has begun to address this question for bacterial transmission by patients with cystic fibrosis [8, 9]. The question of droplet survival duration pertains to droplet size distribution and, on this front, there are discrepancies across the literature which are likely due to the varying measurement methodologies and techniques. Solid impaction with micrometry, a method that is insensitive to smaller droplets, revealed particle sizes from 1 to 2,000 μm [10]. Size distribution analysis using an optical droplet counter [11–13] is reportedly less accurate for particles >2 μm [14] but is useful in the submicron size range. Other previous techniques to measure size distribution include aerodynamic droplet sizer [15, 16], scanning mobility particle spectrometer [15], Andersen six-stage cascade impactor [17], electrical low pressure impactor [18], interferometric Mie imaging [19] and laser diffraction [13, 20]. Aside from the dependency on initial size, the change in droplet size due to evaporation and condensation is also directly related to their chemical composition [21]. Recent findings suggest a tri-modal size distribution during speech or voluntary coughing, with bronchiolar fluid film burst and laryngeal modes both contributing to a distribution peak centred near 1 μm size and with an oral mode yielding a distribution peak centred near 100 μm size due to droplets produced between the epiglottis and the lips [22].

Measurements near the mouth (0.17 m distance) of healthy non-smokers, giving best-effort voluntary coughs, indicate that 99% of expelled droplets are inhalable (<10 μm) [20]. For infected individuals, significant influenza RNA also appears to be contained in droplets with diameters in the respirable size range - 35% of influenza RNA was found in droplets of greater than 4 μm diameter, 23% in droplets of 1 to 4 μm diameter and 42% in droplets of less than 1 μm diameter [23] - and symptomatic subjects appear to emit more particles [18]. However, few results are available for the relationship between droplet size and infectivity for influenza virus.

The viability of airborne pathogens also depends on other factors, including environmental humidity, temperature and the presence of ultraviolet light. The relationship between these factors and infectivity is poorly understood. A recent study using a breathing mannequin and bioaerosol samplers indicated that high relative humidity (RH) was associated with reduced infectivity of influenza virus [24]. Earlier studies using small settling chambers, influenza bioaerosols and guinea pigs found inactivation at high RH [25–28]. Evaporation and particle shrinkage are expected since particles typically enter an environment at lower RH than the respiratory tract [14]. The evaporation process is fast; particles of 10 mm diameter or less typically evaporate in less than 0.5 seconds [21]. At low RH, droplets evaporate more quickly and remain suspended longer compared to droplets generated under conditions of higher RH, thereby increasing the probability of ensuing inhalation [6, 21, 29]. Temperature has also been shown to enhance or interrupt transmission at low (5°C) or high (30°C) temperature, respectively, for all values of RH [28]. Assessment of data in the literature suggested a relationship between absolute humidity (AH) and influenza survival. When extended to a human population level study, negative local daily deviation of AH from its 31 year mean was found to be associated with the start of influenza outbreaks during the winter [30, 31]. Studies on the effect of ultraviolet light irradiation showed avian influenza A (H7N9) virus was inactivated after at least thirty minutes of exposure [32], while adenoviruses appeared to be UV-resistant [33, 34]. The vaccinia virus was found to be less susceptible to ultraviolet radiation at high RH than at low RH [35]. The precise mechanisms through which RH, AH, temperature and UV light exposure affect virus transmission and survival require clarification.

Since the Severe Acute Respiratory Syndrome coronavirus (SARS CoV) outbreak in 2003 [36], the scientific and medical communities, as well as the general public, have gained an appreciation of the public health importance of understanding respiratory virus transmission. Although cough droplet sizes have been characterized, more research is needed to examine cough flows from infected individuals. Much is known about airflow rates during coughing [37–39], including parameters such as Cough Peak Flow Rate (CPFR), Peak Velocity Time (PVT) and Cough Expired Volume (CEV), that is the area under the flow rate versus time curve. A study of 12 female and 13 male subjects showed that the non-dimensional airflow rate (Flow rate/CPFR) versus non-dimensional time (Time/PVT) curve could be defined by two gamma-probability functions based on the medical parameters of CPFR, PVT and CEV that were themselves related to height, weight and gender [40]. Also, a sequential cough was found to be a combination of two single coughs, with the first being approximately the same as a single cough, whilst the second was a scaled down version of the first. Visualizations [40] showed that the airflow direction did not vary greatly amongst the subjects and, although mouth opening remained constant during a cough, there was a considerable variation of area across the subjects with no correlation to other parameters such as height. Their research examined the bulk parameter of flow rate, whilst earlier studies examined the flow field qualitatively using strobe photography [41] or thermal plume imaging by Schlieren [42]. More recently, quantitative analysis of shadowgraph images indicated that this technique requires a significant temperature difference (10°C) between exhaled air and room temperature and that coughs were detectable out to a maximum distance of 0.6 m from the source [43]. Quantitative analysis of high-speed video images of a cough were limited to a similar range whilst also requiring the cougher to expel cigarette smoke as a tracer substance [44], which is problematic to apply in the study of humans with respiratory illness due to the unacceptable possibility of causing harm to participants. Accurate velocity measurements at greater distances in the far-field of a cough require a different measurement technique and approach.

Particle Image Velocimetry (PIV) velocity measurements have been undertaken using an artificial cough flow simulator [45, 46], a thermal mannequin with simulated breathing [47, 48] and healthy human subjects [19, 49–51]. Such measurements have revealed a peak cough velocity of 6 to 22 m/s, with an average of 11.2 m/s [51], but usually mouth area is merely assumed and not measured [50–52]. Kwon *et al*. [53] reported average initial cough velocities of 15.2 m/s (males) and 10.6 m/s (females), with the angle of exhaled air being 38° (males) and 32° (females), although it is doubtful whether this difference in angle is statistically significant. Singh *et al*. [39] found the peak flow rate produced by women to be 60% that of men and Chao *et al*. [19] reported the maximum cough velocity of women to be approximately 77% that of men. On the other hand, VanSciver *et al*. [49] found no significant difference in maximum cough velocity related to sex and weight of the cougher. Furthermore, they noted that, from the fluid dynamic point of view, a cough may be considered as a short-duration transient jet, being notably unlike a very-short-duration jet in which much of the cough would be entrained in a single vortex ring. Their PIV data showed a wide range of maximum cough velocities (1.5 to 28.8 m/s) and that the self-similarity of flow profiles associated with a transient jet was not applicable to coughs, such that it is necessary to develop an envelope of cough profiles rather than attempting to define a “typical” cough. The measurements by Zhu *et al*. [51] showed that some saliva droplets produced during a cough can travel further than 2 m and (using the Lagrangian equation) that the transport characteristics of expelled saliva droplets change with size. Furthermore, a recent study of patients with cystic fibrosis emitting cough aerosols, which were collected with an Anderson Impactor in a wind tunnel of modest cross-sectional area, reported that viable bacteria can travel 4 m from the patient or remain aloft for up to 45 minutes [9]. These findings call into question the “3 feet/1 metre rule” or “6 feet/2 metre rule”, which have been considered to be safe separation distances for preventing droplet transmission [54], and motivate further study of virus-laden bioaerosols and the velocity field at extended distances from the source. Indeed, all previous PIV flow measurements have been taken near the mouth, where velocities are highest, rather than far downstream near the limits of possible person-to-person transmission.

The novelty of the current collaborative research project – the Western Cold and Flu (WeCoF) aerosol study - lies in the fact that the fluid dynamics of the jet aerosols produced by a minimally-confined cough is being examined concurrently with the biological processes associated with virus droplet formation and transmission, using human subjects when they are naturally infected by influenza virus and, again, when they return to health. This is in contrast to previous fluid dynamic studies that have measured the velocity field using artificial aerosol sources or only healthy subjects. Ultimately, the research aims of the present WeCoF project are;

1. to understand the penetration of viral droplets into the ambient environment,
2. to rigorously test the “3 feet/1 metre” and “6 feet/2 metre” rules and
3. to identify host determinants of individuals who emit higher quantities of virus which disperse further,

all of which are important for implementation of future transmission prevention measures. The measured data will also be of use to other researchers who are attempting to develop realistic theoretical [55] or computational fluid dynamics (CFD) [50, 56] models for cough jets/plumes and virus transmission. Such models require reliable modeling of the transport of aerosol droplets and virus particles. In addition, data from human subjects may be used to test simpler models that use the spatial distribution of expiratory aerosols and the viability functions of airborne viruses to estimate exposures to airborne viruses in the indoor environment, where previously such models were based on artificial puff sources, e.g. [57]. This introductory review has covered issues such as cough droplet sizes and the influence of environmental parameters, notably temperature and relative humidity, which will be studied as part of the WeCoF project. However, the present paper focuses on the experimental facility and methods and presents the results from the initial experiments using healthy human volunteer subjects.

### Test facility

**Figure 1:**
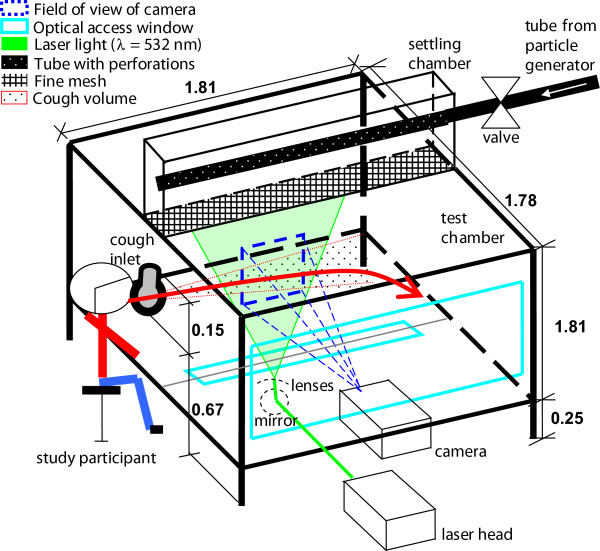
**Diagrammatic layout of the 2 m × 2 m × 2 m FLUGIE cough chamber (all dimensions shown in metres).**

A novel experimental facility (the FLUGIE cough chamber) has been constructed at Western and protocols developed for its use. Biosafety and research ethics approvals have been obtained for studies involving human participants who are naturally-infected with pathogens such as influenza virus. Figure 1 shows a diagrammatic layout of the facility for PIV measurements, with the subject seated outside the chamber. Since the entrainment of ambient air into the cough jet is important to the development of that jet, a solid barrier with only a small opening for the mouth (such as the 5 × 3 cm hole used by [51]) is inappropriate. In the FLUGIE, the opening is pear-shaped such that the participant’s nose and mouth area are unobstructed whilst a cough is directed into the enclosed test chamber. The major vertical axis of the pear-shaped opening is 15 cm high and the base of the opening, where the participant’s chin rests, is 67 cm above the chamber floor. The minor horizontal axis of the pear-shaped opening is 10.5 cm wide. This chamber inlet has a cover, which is only opened when a cough is introduced into the chamber.

The position of the participant’s head is fixed by a chin rest and a forehead rest, such that the angle of the cough is horizontal and consistent over multiple trials. Although it may be argued that a more natural cough would be observed by permitting unrestricted head motion, it is likely that such freedom would permit a significant cough velocity component due to forward translation of the participant’s upper body during the expulsive phase, as well as introduce a greater unpredictability to the cough flow trajectory, which would be problematic for any experimental technique with a limited measurement site or window. From the perspective of achieving the present research aims in a controlled laboratory experiment, it is acceptable to examine the cough velocity produced by pulmonary effort alone.

An open bench set-up [20] may be distinguished from a study of a confined cough but, in essence, all coughs in an indoor setting are the latter type. The salient point is to allow sufficient separation between the cough and all solid boundaries, such that the flow does not exhibit any significant deviation from a cough in fully open surroundings. Even though a participant may be coughing at an open bench towards an open fume hood, the distance from the mouth to the fume hood is of interest, as well as whether the fume hood fan is operating and possibly affecting the cough flow. Hence, a chamber of ample size is preferable as a quiescent environment in which a cough flow may be studied without flow disturbances from uncontrolled surroundings. The internal dimensions (1.81 m length, 1.78 m width and 1.81 m height) for FLUGIE were sized for separation distances to be greater than participant mouth diameter by more than an order of magnitude. At the measurement window, the separations decrease to less than an order of magnitude due to the lateral spreading of the cough flow with distance from the mouth, yet remain several times the lateral extent of the cough. The test chamber is raised by 0.25 m above the laboratory floor and mounted upon casters to allow measurement at various streamwise positions.

**Figure 2:**
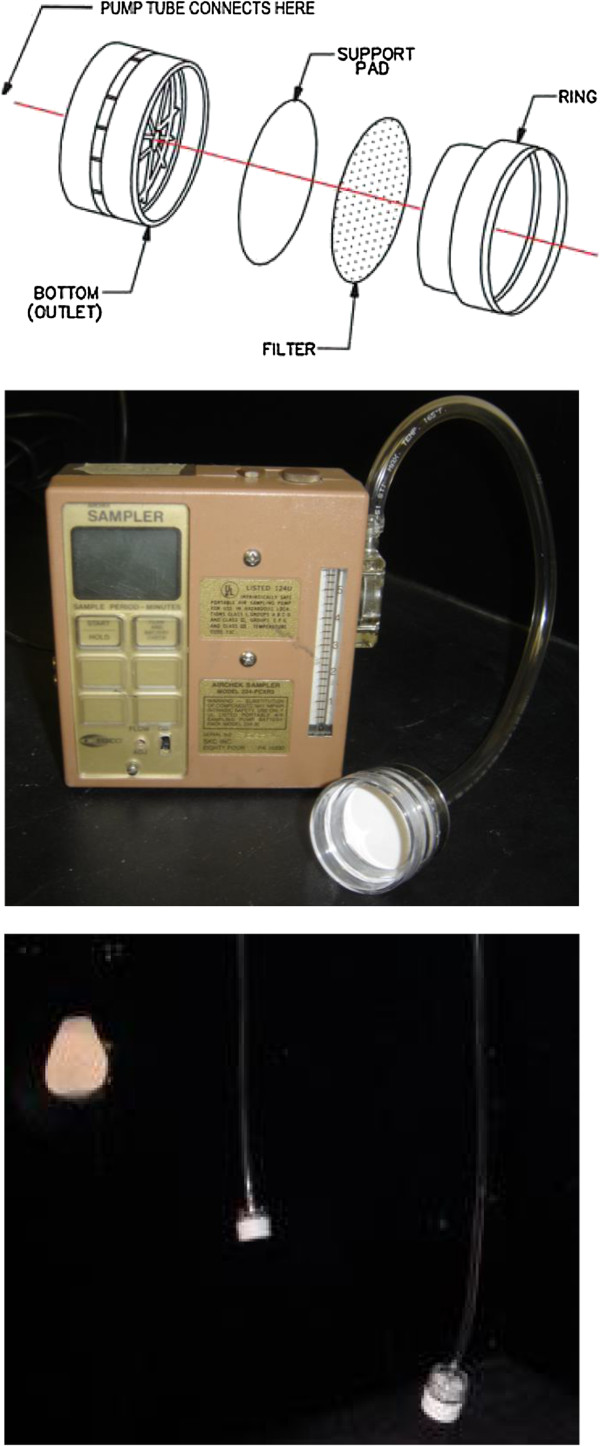
**Diagram of the bioaerosol sampling cassette assembly (top), photograph of a cassette attached to a sampling pump (centre) and photograph of the cassettes in operation in the cough chamber (bottom).**

In order to quantify the viral content of the aerosols produced during coughs, particles are sampled by collection onto polytetrafluoroethylene (PTFE) membrane filters of 1.0 μm pore size and 37 mm diameter. As shown by Figure 2, a filter and cellulose support pad is held between a clear styrene cassette ring and bottom. The open-faced cassettes are suspended from the chamber roof, on the chamber centreline, at two distances from the cough inlet (0.5 m and 1.0 m). The constant-flow air sampling pumps (SKC Inc., Airchek 224-PCXR3) are operated at a flow rate of 4000 ± 40 mL/min. Expelled pathogens are collected upon the membranes with the assistance of the air flow samplers.

The identity of the pathogen acquired by each study participant is confirmed by asking for a self-collected mid-turbinate swab (MTS) and these specimens are interrogated by multiplex polymerase chain reaction (multiplex-PCR) assay for a panel of respiratory viruses (RVP Fast, Luminex). The viral content from the membranes is quantified using a virus-specific monoplex quantitative real-time PCR assay and calculated using quantitative curves and number of litres of air sampled.

### Methods

#### Particle Image Velocimetry of coughs

Separate measurements are performed to quantify the cough flow field. Optical access areas into the test chamber are outlined in light blue in Figure 1. A beam of green light (532 nm wavelength) horizontally emitted from the laser head (120 mJ, Nd:YAG crystal) is re-directed to vertical by a 45°-angled mirror and expanded into a narrow light sheet (~1 mm thickness) with cylindrical and spherical lenses. The light sheet enters the test chamber through a glass window in its floor, and illuminates a centreline plane from the test chamber floor to the test chamber roof. The Vieworks VA-4 M32 camera is a charge-coupled device (CCD) with a resolution of 10.0 pixels per mm and a sensor array of 1,752 pixels by 2,336 pixels, where the longer side is oriented vertically for this experiment. The camera is focused upon the light sheet at the chamber centreline and optical access is through a glass window on a chamber wall.

The test chamber is seeded with titanium dioxide particles (rutile mineral form). The product specifications indicate a particle size distribution ranging from 0.15 to 0.47 μm, where 69% of the particles are in the 0.34 to 0.43 μm size bin and 29% of the particles are in the 0.27 to 0.34 μm size bin. The titanium dioxide (TiO_2_) powder is dried in a vacuum-oven, stored in a vacuum container to minimize clumping and aerosolized using a custom-crafted version of the Pitt 3 aerosol generator [58]. This device consists of a cylindrical drum with small inlet and outlet ports near its bottom and top ends, respectively. The drum is filled with TiO_2_ powder, which is carried up and out of the drum by the flow driven by a 30 kPa air line attached to the inlet port. The drum is placed on top of a loudspeaker, which generates sound waves to vibrate and break up the powder. From the outlet port of the aerosol generator, the aerosolized particles enter a settling chamber mounted on top of the test chamber through a tube with perforations. The FLUGIE settling and test chambers are separated by a fine mesh, which permits TiO_2_ particles, under the action of gravity and local airflow, to gently enter the test chamber along its centreline. The cough jet generated by the participant disturbs the TiO_2_ particles which are imaged to obtain quantitative information of the flow field.

Thus, this setup achieves an intersection of the tracer particles, the light sheet illuminating the tracer particles, the focused field-of-view of the camera recording the illuminated tracer particles and the cough flow, over a sizable region of space and time (400 cm^2^ and 5 s, respectively). A pulse generator (Berkeley Nucleonics Corporation, Model 555-4C) is used to control the timing and synchronizing of the laser and camera. Image pairs are captured at a rate of 16.7 Hz, from which instantaneous velocity fields are calculated using commercial software (TSI Incorporated, Insight3G) that cross-correlates the image pairs. In the tests reported here the field-of-view of the camera is centred at 1.00 m from the cough inlet. For each study participant, following the aforementioned cough droplet sampling, PIV is performed for another three independent single coughs with a settling time of 30 s between each cough.

A preliminary study involving healthy individuals has been carried out in order to assess the performance of PIV for measuring the far-field region of this transient and turbulent air flow. In addition, this work has provided the framework for a more extensive campaign that will encompass a statistically significant cohort. The velocity fields associated with the coughs from 12 healthy young adults were quantified at a distance of 1 m away from the mouth. This limited study with healthy volunteers leads into the recruitment and study of individuals who are naturally-infected with influenza virus. Written informed consent was obtained from all participants. The University of Western Ontario Research Ethics Board for Health Sciences Research Involving Human Subjects (HSREB) reviewed and approved this study. The HSREB is registered with the U.S. Department of Health & Human Services under the registration number IRB 00000940.

#### Participant recruitment

In an ongoing pilot study at a university student health clinic (Student Health Services at Western University), a small cohort of undergraduate students, who were naturally-infected with influenza, are being referred by clinicians to the WeCoF aerosol study. Written informed consent is being obtained from all participants in this ongoing study. The HSREB reviewed and approved this study.

#### Disinfection measures

A germicidal lamp, which produces continuous light in the Ultra-Violet B range, is used to disinfect the test chamber between study participants. In addition, an outlet has been retrofitted with a HEPA filter through which chamber air can be withdrawn to further reduce the risk of viral contamination between subjects. The experiments are repeated several weeks later, with the same participants, after recovery from the respiratory illness to permit an assessment of the differences in the coughs between an infected and a healthy person.

### Discussion of preliminary results

**Figure 3:**
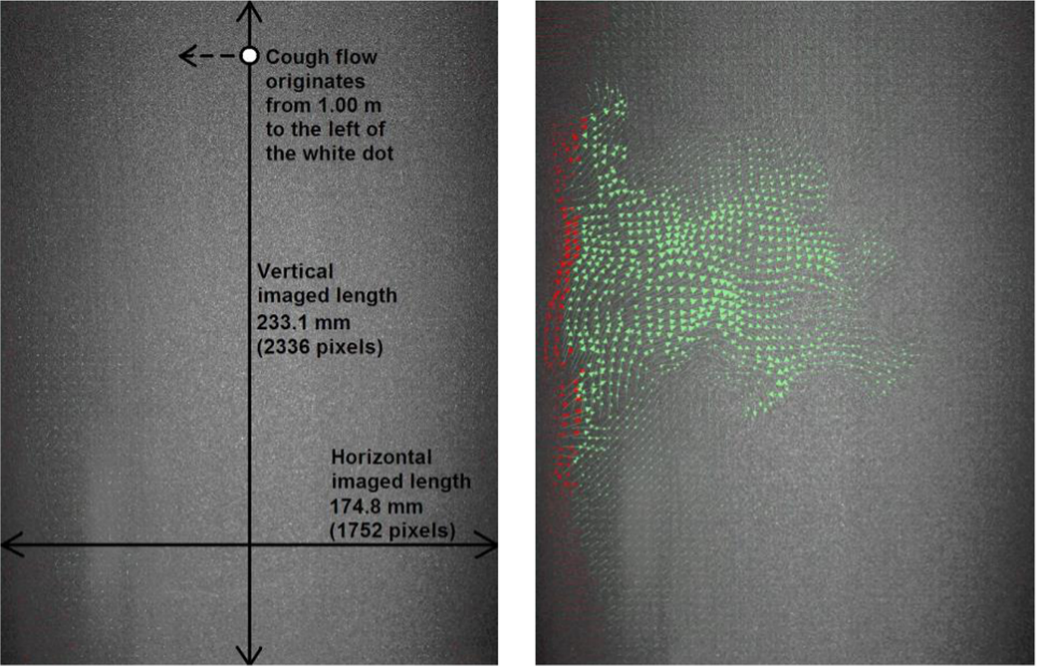
**Field of view for the cough airflow measurements (left) and an example of an instantaneous vector field obtained during a cough (right).**

Preliminary experiments have been carried out involving 12 healthy volunteer participants (9 male, 3 female, ages 20 to 32). Since they were healthy, cough airflow measurements using PIV were conducted without viral aerosol sampling. Each participant produced 3 coughs with the PIV system recording image pairs prior to, during and after the cough. These were then processed to yield instantaneous velocity vector arrays within the field of view. Figure 3 (left) shows that the field of view was located 1.00 m downstream of the entry to the FLUGIE cough chamber and encompassed a region of 174.8 mm in the streamwise direction, centred at the 1.00 m location. The field of view extended over 233.1 mm vertically and was below the level of the cough to take into account the fact that, even if the cough was initially directed horizontally forward by the study participant, most of the coughs had drifted downwards at the 1 m location, contradicting the importance of buoyancy in a proposed model based on visualizations out to 0.70 m from a participant in an open laboratory setting [55]. An example of a processed vector field is shown in Figure 3 (right), where the green arrows are vectors that have passed validation by the Insight3G processing software. Red vectors are spurious values, which were typically attributable to the reduced light sheet intensity at the edges of the field-of-view, whose size approached the upper limits of this PIV equipment. Based on the selected interrogation window size (i.e. the area from which a vector was calculated), the maximum possible number of vectors in each processed image was 3,816.

**Figure 4:**
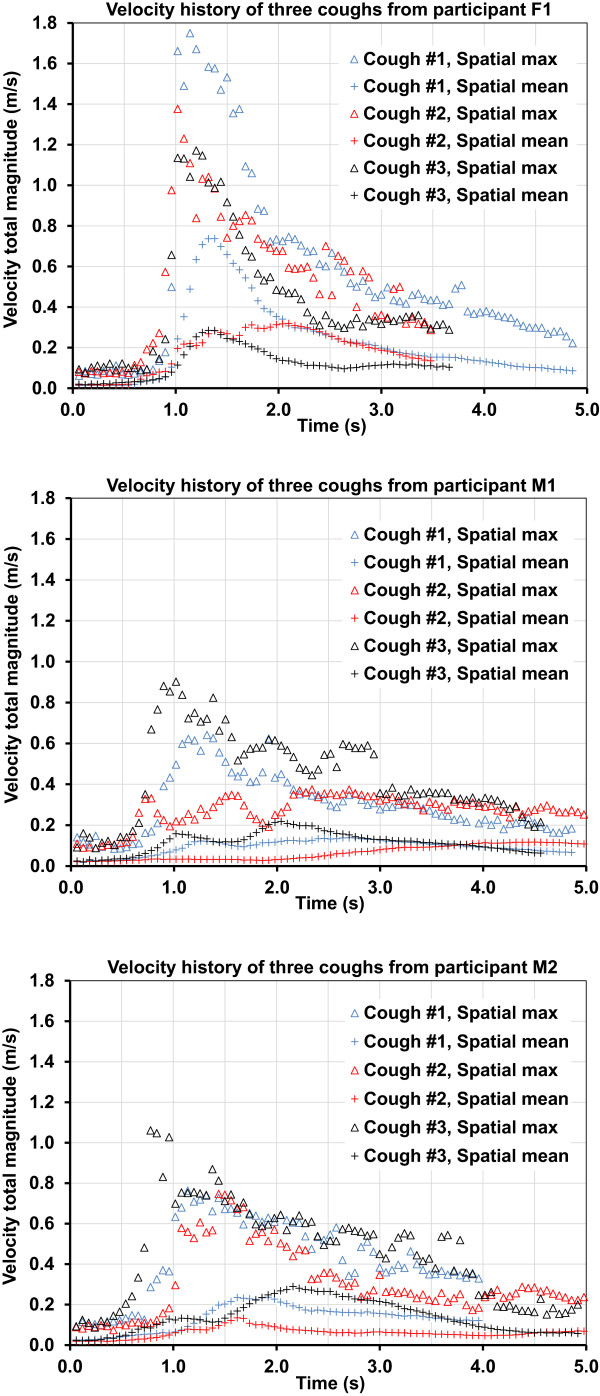
**Time-histories of three coughs from a female (F1) and two male (M1 and M2) participants**.

It should be noted that the 12 participants had their coughs recorded on different days. For 3 participants, the results were poor due to low particle seeding levels and low numbers of validated velocity vectors (only about 0.8% of the total number of vectors were considered to be accurate, representing approximately 30 vectors per image). Three other participants were recorded in another session and showed the best results in terms of percentage of validated vectors, with values around 90% (their data are shown in Figure 4). The other 6 subjects were recorded on a separate day and showed acceptable validation levels in the 70 to 80% range. The findings for each of the three coughs by each of the twelve participants are given in Table 1, where the first character in the participant identifier (ID) indicates participant gender (F for female and M for male) and the second character is an integer index for each individual of that gender. The magnitude of each vector in each image pair was extracted with this quantity averaged (i.e. spatially) over the entire number of such validated vectors to produce a value representing the average air velocity within the field of view and the maximum mean value, occurring in each individual cough event, is shown in Table 1. A representative measure of processed data quality is included, together with observations. Furthermore, the peak value of velocity from each image pair was extracted and plotted against time for that cough.

The time-histories of spatially-averaged and peak velocity values for three subjects are shown in Figure 4. It may be seen that, in all cases, the motion of the cough through the field of view, located a metre away from the cougher, is clearly defined with an initial rapid increase of velocity followed by a slower decay. It is also evident that there is no single characteristic shape for a cough velocity profile and, thus, it is necessary to define an envelope of cough profiles based on the measurement and analysis of a larger number of coughs than those examined in these initial trials.

**Figure 5:**
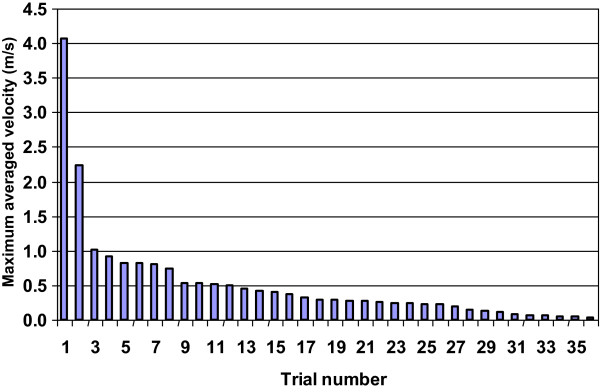
**Variability of maximum spatially-averaged velocity magnitude across the 36 trials.**

**Figure 6:**
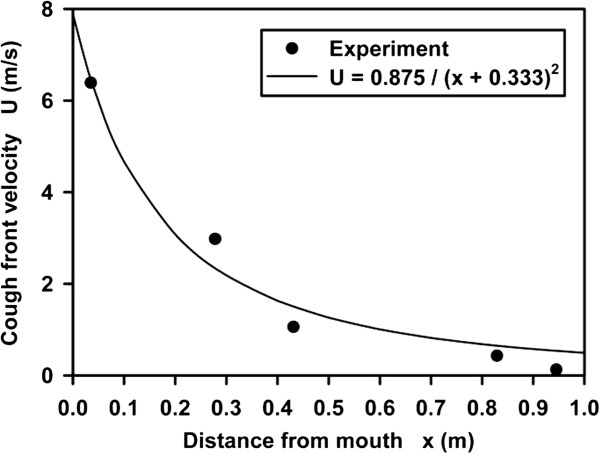
**Example of the decay of cough front velocity with distance from mouth.**

As would be expected from the limitation on the PIV window size and the variable physical traits of study participants, it was found that there was considerable variation in location and strength of each cough, with some coughs missing most of the imaged field of view entirely. The distribution of values of the spatially-averaged maximum velocity magnitude is illustrated in Figure 5. The average value across all 36 coughs is 0.52 m/s, but with the data from the three poor quality experiments removed, the average across the remaining 27 tests is 0.41 m/s. These values indicate that there is significant air motion during a cough, of the order of 0.5 m/s, at a location as far away as 1 m from the person who is coughing. During the set-up phase for this preliminary study, a single volunteer produced 5 coughs, with a 180-mm-wide PIV field being located at different distances from the mouth for each cough. The participant attempted to produce a series of coughs each of the same strength. Although the quality of the resulting vector field was not sufficient to provide quantitative data, it was possible to identify the arrival of the cough front at each location and, thereby, estimate the velocity of the cough front at the centre of each field of view. The results are shown in Figure 6, illustrating the rapid decrease in velocity in the near-field of the mouth, as would be expected. At 1 m from the mouth the cough front velocity has a magnitude in agreement with the average of the spatially-averaged maximum velocity magnitude from our preliminary study with 12 healthy participants. Note that the fitted curve is based on an approximation of a linear growth of the cough jet diameter with distance from the mouth.

**Table 1:** **Summary of 36 measured coughs from 12 healthy participants**

ID	Cough #	Max Mean velocity (m/s)	Mean # of Valid vectors (%)	Observations
F1	1	0.737	89.5	Strong; wide; partially high
F1	2	0.319	90.5	Strong; wide
F1	3	0.285	91.3	Partially high
F2	1	0.266	84.6	In field of view
F2	2	0.064	81.8	Missed, too low
F2	3	0.254	81.8	In field of view then low
F3	1	0.821	74.2	Strong; wide
F3	2	0.911	74.9	Strong; in field of view
F3	3	0.239	74.3	Strong; horizontal flow
M1	1	0.138	90.2	Strong; partially high
M1	2	0.117	90.2	Weak; wide; multiple jets
M1	3	0.218	90.0	In field of view; intermittent
M2	1	0.236	91.2	In field of view
M2	2	0.135	90.7	Low
M2	3	0.289	90.1	Strong; wide
M3	1	2.226	79.0	Sharp front; wide
M3	2	0.826	77.5	Violent; wide
M3	3	1.017	76.7	Sharp front; wide; intermittent
M4	1	0.196	0.8	Low seeding; spurious vectors
M4	2	0.371	0.7	Low seeding; spurious vectors
M4	3	0.798	0.7	Low seeding; spurious vectors
M5	1	4.065	0.7	Low seeding; spurious vectors
M5	2	0.533	0.7	Low seeding; spurious vectors
M5	3	0.421	0.7	Low seeding; spurious vectors
M6	1	0.532	73.2	Strong; wide
M6	2	0.498	72.2	Wide; some spurious vectors
M6	3	0.449	71.8	Turbulent; in field of view
M7	1	0.516	0.7	Low seeding; spurious vectors
M7	2	0.407	0.7	Low seeding; spurious vectors
M7	3	0.274	0.8	Low seeding; spurious vectors
M8	1	0.074	74.3	Weak; partially low
M8	2	0.041	72.1	Weak; partially low
M8	3	0.047	73.3	Best for M8; intermittent
M9	1	0.031	74.7	Very weak and low
M9	2	0.068	75.9	Weak; partially low
M9	3	0.231	74.1	Strong; wide

### Conclusions

A novel experimental facility – the FLUGIE – has been designed to study the far-field aerodynamics of human coughs produced by subjects naturally-infected with respiratory viruses, together with measurement of the viral content of the droplets produced by those coughs, in order to quantify the factors relating to person-to-person airborne transmission of virus. A preliminary study involving 12 healthy individuals has been carried out in order to quantify the strengths of their coughs at a distance of 1 m away from the mouth. The velocity fields were measured using Particle Image Velocimetry and the results indicate an important finding, namely that there is significant air motion during a cough, of the order of 0.5 m/s, even at a location as far away as 1 m from the person who is coughing.
